# Oral neutrophil quantitation in patients undergoing elective cardiopulmonary bypass

**DOI:** 10.1186/cc10622

**Published:** 2012-03-20

**Authors:** ME Wilcox, P Perez, C DosSantos, M Glogauer, E Charbonney, A Duggal, S Sutherland, G Rubenfeld

**Affiliations:** 1 University of Toronto, Canada; 2 Sunnybrook Health Sciences Centre, Toronto, Canada; 3 St Michael's Hospital, Toronto, Canada

## Introduction

Recent research suggests that the oral cavity may provide an early opportunity to monitor the innate immune system; an oral rinse assay was found to be a reliable predictor of bone marrow engraftment and neutrophil recovery in patients undergoing bone marrow transplantation [1]. Multiorgan failure may be mediated by neutrophil extravasation and aggregation [2] in highly inflammatory states, such as cardiopulmonary bypass (CPB). The objective of this novel pilot study was to determine whether the kinetics of oral neutrophil recovery post-CPB surgery reflect systemic immune activation.

## Methods

Samples [3] from four-quadrant mucosal swabs and oral cavity rinses were obtained from 41 patients undergoing on-CPB elective cardiac surgery preoperatively (t-1) and postoperatively upon arrival to the CVICU (t0), at 12 to 18 hours (t1), and on day 3 (t2). Oral neutrophil counts (/ml) were determined by hemacytometry and validated by an electronic cell counter. Concurrent blood samples were collected for measurement of IFNα, interleukins (IL-1β, IL-6, IL-8 and IL-10), chemokine C-C motif ligand 4 (CCL-4) and Th1 and Th2 cytokines using a 10-plex human cytokine mediator panel. Continuous variables were summarized with means (standard deviation). Preoperative and postoperative oral neutrophil counts were compared using paired *t *tests.

## Results

Patients were 65 (10.6) years old; 78% male; 51% had significant co-morbidities (25% diabetes); 54% took a statin; APACHE II score was 22 (4.4); and multiorgan dysfunction score (MODS) was highest on hospital day 1 (6.2; 2.2). Mean delta oral neutrophil count by oral swab (between t-1 and t0) was 1.7 × 10^6 ^(2.0 × 10^6^). A significant difference was seen in the absolute neutrophil counts (oral swab) between t-1 (1.7 × 10^6 ^(1.3 × 10^6^)) and t0 (3.4 × 10^6 ^(2.7 × 10^6^); *P *< 0.001), but not between t-1 and t1 (2.0 × 10^6 ^(1.7 × 10^6^); *P *= 0.14) or t2 (6.6 × 10^5 ^(1.1 × 10^6^); *P *= 0.14). Similar results were obtained by oral cavity rinse.

## Conclusion

An oral swab assay has the potential to provide rapid, risk-free, and early data on neutrophil activation and chemotactic defects in response to CPB, obviating the need for invasive sampling. This method could provide a new perspective on the systemic inflammatory response in surgery, traumatic injury, burns, and sepsis.
